# Facilitation of Tumor Stroma-Targeted Therapy: Model Difficulty and Co-Culture Organoid Method

**DOI:** 10.3390/ph18010062

**Published:** 2025-01-08

**Authors:** Qiu-Shi Feng, Xiao-Feng Shan, Vicky Yau, Zhi-Gang Cai, Shang Xie

**Affiliations:** 1 Department of Oral and Maxillofacial Surgery, Peking University School and Hospital of Stomatology & National Center of Stomatology & National Clinical Research Center for Oral Diseases & National Engineering Research Center of Oral Biomaterials and Digital Medical Devices, 22# Zhongguancun South Avenue, Haidian District, Beijing 100081, China; qiushifeng163@163.com (Q.-S.F.); bdkqsxf@163.com (X.-F.S.); 2 Division of Oral and Maxillofacial Surgery, Columbia Irving Medical Center, New York City, NY 10027, USA; makjamajnam@gmail.com

**Keywords:** stroma mimicry model, patient-derived tumor organoid, co-culture, stroma-related cell, stroma-targeted therapy

## Abstract

**Background:** Tumors, as intricate ecosystems, comprise oncocytes and the highly dynamic tumor stroma. Tumor stroma, representing the non-cancerous and non-cellular composition of the tumor microenvironment (TME), plays a crucial role in oncogenesis and progression, through its interactions with biological, chemical, and mechanical signals. This review aims to analyze the challenges of stroma mimicry models, and highlight advanced personalized co-culture approaches for recapitulating tumor stroma using patient-derived tumor organoids (PDTOs). **Methods:** This review synthesizes findings from recent studies on tumor stroma composition, stromal remodeling, and the spatiotemporal heterogeneities of the TME. It explores popular stroma-related models, co-culture systems integrating PDTOs with stromal elements, and advanced techniques to improve stroma mimicry. **Results:** Stroma remodeling, driven by stromal cells, highlights the dynamism and heterogeneity of the TME. PDTOs, derived from tumor tissues or cancer-specific stem cells, accurately mimic the tissue-specific and genetic features of primary tumors, making them valuable for drug screening. Co-culture models combining PDTOs with stromal elements effectively recreate the dynamic TME, showing promise in personalized anti-cancer therapy. Advanced co-culture techniques and flexible combinations enhance the precision of tumor-stroma recapitulation. **Conclusions:** PDTO-based co-culture systems offer a promising platform for stroma mimicry and personalized anti-cancer therapy development. This review underscores the importance of refining these models to advance precision medicine and improve therapeutic outcomes.

## 1. Introduction

The academic consensus has acknowledged that tumors are not merely epithelial cell clusters that receive carcinogenesis induction, but are complicated organs where neoplastic cells and tumor stroma co-exist and co-evolve [1]. Supported by Stephen Paget’s “seed and soil” hypothesis in the 1880s [2,3,4,5], tumor stroma, the non-oncocyte composition [6], exerts sophisticated yet underestimated impacts on tumorigenesis [7,8], cancer stemness [9,10], cell metastasis [11], and drug resistance [12]. It also inherently determines the immediate niche for tumor tissues [13,14,15], resulting in obvious heterogeneities between lesions, periods, individuals, and tumor types [16,17]. Thus, research on tumor stroma, specifically on its intrinsic properties [18,19], mutual interaction with cancer cells [20], and adaptive responses to therapeutic intervention [21], refutes the notion that efforts for anti-cancer approaches which neglect non-oncocyte components are insufficient.

The overlooking of tumor stroma in cancer regimen development can be principally attributed to challenges in stroma mimicry modeling, which can be briefly attributed to the dynamic signature in tumor stroma. Over the past several decades, assorted models, including two-dimensional (2D) cell lines [22], xenograft models [23], and clinical samples [24], have demonstrated their abilities in anti-stroma scheme development. Yet, specific deficiencies have been noticed. For instance, the absence of cell–cell/cell–extra cellular matrix (ECM) interactions in 2D cultures leads to disappointing fidelity [25,26,27]. For in vivo models, challenges also include prolonged incubation periods, low throughput models, interspecies differences, and strict ethical constraints [28,29]. Evidently, a more practical preclinical model is imperative for achieving specific advancements in stroma-targeted research [25,30].

PDTO, a three-dimensional spheroid [31] generated from tumor tissues or cancer-specific stem cells within tumor tissues [32], has gained phenomenal popularity in therapy assay and drug screening [33,34,35]. Despite the self-assembly mechanism [36,37] that potentially integrates non-cancer components into primary PDTO, the Hayflick limit in stroma-related cells [38] has predicted the transience of tumor stroma in the organoid. However, this can be persistently maintained by artificial co-culture with exogenous stroma-creating cells [39,40,41]. Numerous previous studies have verified that the co-culture system not just optimizes PDTO as a more authentic platform [25], but highlights heterogeneity at the tumor stroma level [42]. This, in turn, promotes personalization and precision in the translation of stroma-targeted therapy [25,43].

**Figure 1:**
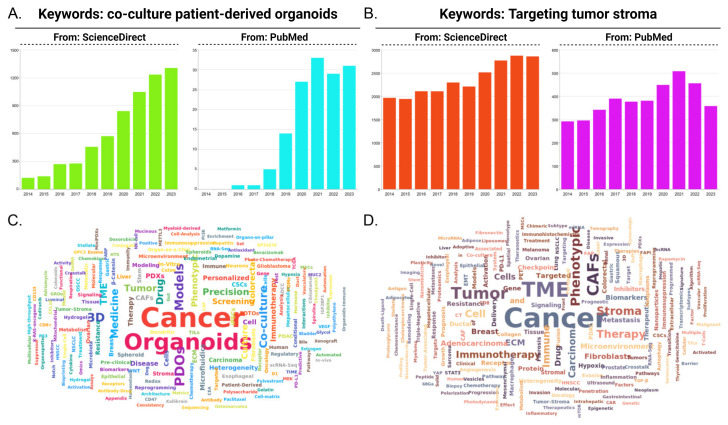
Research trends focusing on co-cultured PDTO and stroma-targeted therapy. (**A**) The number of published studies in the last decade. Search terms “co-culture” and “patient-derived organoids” were searched on academic engines, including ScienceDirect and Pubmed. (**B**) The number of published studies in the last decade focusing on “targeting tumor stroma”. Academic research engines including ScienceDirect and Pubmed were screened. (**C**) Pubmed was utilized to search articles published in last three years, with search terms of “co-culture” and “patient-derived organoids”. Keywords of filtered studies underwent analysis after prepositions were removed. (**D**) Pubmed was utilized to screen articles published in last three years, with search term of “targeting tumor stroma”. Keywords of filtered studies underwent analysis after prepositions were removed.

The last decade, as depicted in Figure 1, has witnessed the mobilization of stroma-targeted cancer therapy [24,44,45,46], along with the maturation of co-culture techniques for tumor organoids [47,48,49,50]. In this review, we discuss challenges in stroma mimicry modeling and summarize relevant models in stroma-targeted therapy development, as well as the utilization of co-culture PDTO in tumor stroma studies. Brief comparisons regarding classic models and co-culture PDTO are also made. In summary, the stroma recapitulation capability of co-culture PDTO for anti-cancer regimen evaluations has been demonstrated.

## 2. Challenges in Tumor Stroma Mimicry Modeling

### 2.1. Intricate Stroma Composition

**Figure 2:**
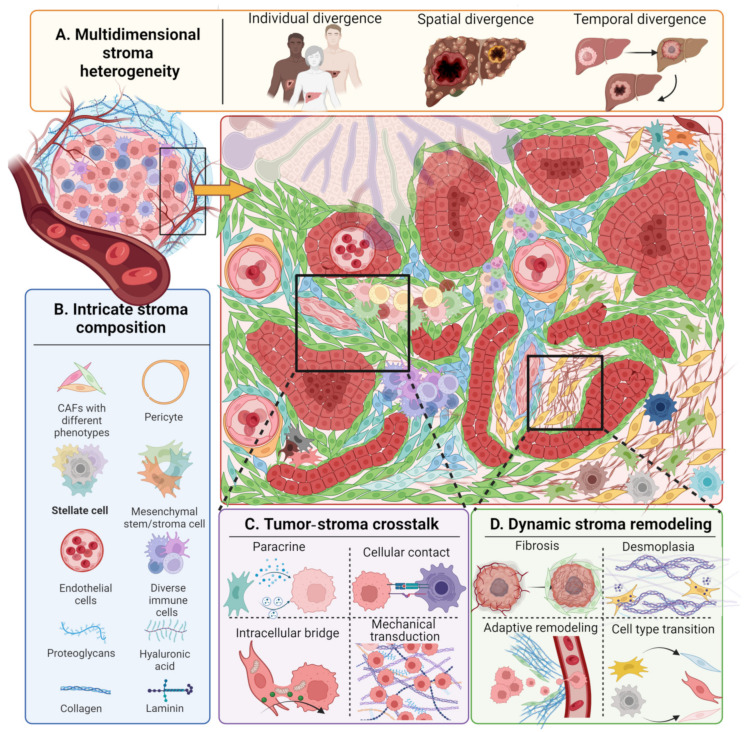
Challenges lying in stroma-targeted modeling. (**A**) Stroma heterogeneities in different aspects, including individual divergence, spatial divergence, temporal divergence, etc. (**B**) Intricate stroma compositions exponentially magnify the complexity in models, especially in vitro and ex vivo. (**C**) Versatile performances of tumor–stroma crosstalk, both in approaches and bio-behaviors. (**D**) Dynamic stroma remodeling, adapting to different TME or certain malignant event.

As shown in the Figure 2, the complexities of stroma ingredients and their dynamic transition correspond with complications in model design. The oversimplification of elements in models distracts from stroma reflection fidelity, while the lack of simplification leads to excessive interferences beyond the targeted stroma element, which curtails the model’s representativeness, and eventually defocuses research [51].

#### 2.1.1. CAFs

As their name implies, cancer-associated fibroblasts represent mesenchymal cells with a typical spindle-shape morphology and planar polarity potential [52]. They survive within tumor tissues or at the invasive edge, and are associated with tumor progression [53]. CAFs receive hyper-activation by cytokines and metabolites from oncocytes and stroma [54], including lactic acid and its derivatives, transforming growth factor-β (TGF-β), epidermal growth factors (EGFs), bone morphogenic proteins (BMPs), fibroblast growth factors (FGFs), etc. [55]. Meanwhile, the positive feedback mechanism in over-activated CAFs incurs more fibroblast recruitment [56]. For instance, Linares et al. reported the activation of tumor-secreted lactate-mediated CAF by decreased p62 expression through NAD reduction, indirectly verifying the CAF-targeted role of olaparib [57]. In another case, an oncocyte-derived extracellular vesicle (EV) led to pro-inflammatory CAF activation in oral squamous cell carcinoma (OSCC) [58].

Instead of a homogeneous cell population, CAFs experience subtype division, based on different criteria, due to different cell origins and assorted inducing factors [59,60]. Luo et al. divided CAFs into three major subpopulations that comprise cancer-associated myofibroblasts, inflammatory CAFs, and adipogenic CAFs, as well as three minor subpopulations that comprise endothelial–mesenchymal transition CAFs, peripheral nerve-like CAFs, and antigen-presenting CAFs [61]. Heterogeneities in TME, location, biofunction, and gene signature all contribute to pan-cancer CAF analysis, which involves 10 solid cancer types based on single-cell RNA sequencing (scRNA-seq) datasets. The proposed mechanism underlying distinctive CAF differentiation includes the antagonism of interleukin-1 (IL-1)-induced JAK/STAT signaling [62], smad3-mediated macrophage–myofibroblast transition [63] and hedgehog (HH) pathway regulation [64].

However, the tumor-restraining effects of specific CAF biofunctions, including immune defense through immunomodulatory cytokines recruitment [65,66] and mechanical restriction through type I collagen accumulation [67], deserve equal attention [52].

As the most abundant cellular composition of the stroma, CAFs are relatively convenient to isolate and incubate during stroma mimicry modeling [59]. However, the lack of high-specific biomarkers for CAFs, especially CAF subsets, hinders the advancement of CAF-targeted therapy development [56]. Additionally, the unexpected and often undetected subpopulation switch of CAFs in in vivo models also diminishes the credibility of the results.

#### 2.1.2. MSC

Initially isolated from bone marrow, mesenchymal stem cells (MSCs), which are versatile multi-potential stem cells, have been found in various solid tumors [68,69,70,71]. In contrast to the predominant tumor-promoting role of CAFs, the impacts of MSC–tumor communication appear to be controversial, marked by their multifaceted influences [72,73]. For instance, Atiya et al. reported that CD10^−^ MSCs supported ovarian clear cell carcinoma (OCCC) via interruption of iron chelation [74]. Similarly, as verified by Zarubova et al., Rac-dependent cell contact between MSCs and less mobile oncocytes is pivotal in tumor cell clusters (CTCCs) formation, and subsequent cancer dissemination [75]. More pernicious behaviors of MSCs consist of accelerating proliferation, fostering therapeutic resistance, promoting immune suppression and evasion, enhancing tumor-homing capability, and promoting angiogenesis [76,77,78,79]. On the flip side, Melzer et al. observed spontaneous hibernation in breast cancer triggered by proactive fusion of MSCs and neoplastic cells [80]. A parallel phenomenon has been verified in ovarian cancer [81]. The equivocal role of MSCs renders them an increasingly prominent therapeutic target or tool by artificially inducing or magnifying their tumor-constraining role [82]. Based on tumor-homing characteristics, for instance, MSCs and their EV have been engineered as drug delivery vehicles, which have been applied by McKenna et al. in oncolytic immunotherapy to augment the efficacy of chimeric antigen receptor T-cells (CAR-T) therapy [83,84].

Not confined to CAFs, MSCs possess other differentiations, including osteoblasts, chondroblasts, and adipocytes, all of which have been identified in tumor tissue. As proof, osteogenesis driven by the betaglycan–Wnt5a circuit is apparent within bladder cancer [85], while exosomes secreted by adipocytes in breast cancer stimulate tumor growth via Hippo signaling activation [86]. Generally, it seems that highly differentiated cells that are derived from MSCs contribute to tumor expansion.

#### 2.1.3. Other Cells

Stellate cells are capable of mutual interactions with CAFs and producing collagenous stroma [87]. This dynamic interplay endows stellate cells with tumor-promoting effects, similar to those of the CAFs [88]. For instance, hepatic stellate cells can awaken breast neoplastic cells from natural killer (NK) cell-mediated dormancy, via the activation of the CXCL12-CXCR4 axis [89]. Additionally, pancreatic stellate cells can facilitate the perineural invasion of pancreatic cancer, through the HGF/c-Met pathway [90].

Pericytes, cells located between the basement membrane and vascular endothelial cells, are responsible for capillary regulation to maintain microcirculation stability in tissue [91]. While, in tumor tissue, the disruption of pericytes indicates active but deficient angiogenesis, and hypoxic TME. Destruction of the vascular barrier and a higher risk of tumor metastasis through the circulatory system is also seen [92]. For example, pericytes from hepatocellular carcinoma (HCC) and non-small cell lung cancer (NSCLC) take part in vibrant glycolysis triggered by hexokinase 2, detrimentally impacting vessel support function via the ROCK2-MLC2 axis [93].

Due to tumor-specific distribution, the paucity of specific biomarkers and the fragility of cell activity, it is difficult to identify, separate and sustain the long-term incubation of pericytes, forming the first barrier for relative modeling.

#### 2.1.4. ECM

The distinctive mechanical transduction provided by the ECM, the fundamental scaffold of cellular components [94], primarily depending on stiffness regulation [95] through collagen and elastin synthesis, arrangement, and cross-linking [96], establishes the paramount role of ECM in tumor–stroma interactions. An increase in ECM stiffness can promote HCC proliferation via exosome release and Notch signal activation [97], drive breast cancer motility through thrombospondin-1 [98], and accelerate ovarian cancer progression via the Src gene and the RhoA/ROCK pathway [99]. In addition, ligand accessibility, cellular adhesion, microenvironment topography, and outside-in forces regulated by the ECM also determine various malignant behaviors [100]. However, the mechanical environment created by ECM can be hardly recapitulated by 2D in vitro models because of its innate structure. Meanwhile, although 3D models supported by matrigel, hydrogel, or the acellular matrix can partially mirror stress distribution [101,102,103], artificial materials lack dynamic remodeling capabilities, and the component identification of the natural matrix also poses a formidable challenge.

### 2.2. Complex Tumor–Stroma Crosstalk

Already on the radar of pharmacologists, the intimate crosstalk between tumors and stroma, an accomplice of cancer deterioration, holds clinical significance as an advanced target in anti-cancer therapy [104]. It has been indirectly proven by diagnostic and prognostic roles of crosstalk-related parameters, including tumor–stroma ratio (TSR) [105], metabolic-tumor-stroma score (MeTS), and tumor–stromal interfacial qualification [106].

#### 2.2.1. Crosstalk Approaches

Diverse crosstalk approaches have been observed in tumor stroma. For instance, Liu et al. proposed that direct cell–cell contact from CAFs promotes tumor organoid growth [42], while Ma et al. identified the paracrine of Wnt5A as a crucial mechanism for conferring stemness to bladder cancer cells [107]. In addition, miR-522 contained in CAF exosomes were observed to inhibit ferroptosis in gastric cancer by impeding lipid ROS accumulation [108]. Furthermore, EVs from CAFs loaded with Annexin A6 were found to bestow drug resistance to gastric cancer by inhibiting FAK-YAP signaling [109]. Meanwhile, the chemical alternations in the TME, mediated by the metabolic reprogramming of CAFs [110], such as heightened glycolysis [111], glutamine metabolism [112], and fatty acid catabolism [113], were noted to facilitate the progression of breast cancer. Multifarious crosstalk approaches make researchers cautious when selecting model types based on their hypothesis.

#### 2.2.2. Phenotypes Influenced by Crosstalk

The influence of tumor–stroma crosstalk on malignant cells can be manifested in a spectrum of phenotypes. For instance, when investigating the crosstalk mediated by CAF-derived exosomes, Zhao et al. reported metabolic reprogramming in oncocytes via the micropinocytosis mechanism [114], while Qi et al. suggested drug resistance via ferroptosis inhibition through ACSL4 sponginess [115]. Concurrently, CAF–oncocyte crosstalk via exosomes has been associated with a range of other outcomes, including stemness enhancement in colorectal cancer through β-catenin pathway activation [116], epithelial–mesenchymal transition (EMT) acceleration in lung cancer via Snail1 transport [117], proliferation promotion in NSCLC via PTEN downregulation [118], and migration encouragement in breast cancer via ubiquitin-specific peptidase 28 binding [119].

#### 2.2.3. Mutual Reaction

The communication between tumors and stroma is a reciprocal process. Built on their work on CAF-mediated crosstalk, Li et al. reported that methylmalonic acid, an oncometabolite secreted by aggressive oncocytes, activated the CAF phenotype of normal fibroblasts within TME. In the meantime, CAF-origin exosomes loaded with IL-6 were identified as an accelerator of the progression of lung squamous cell carcinoma (LSCC) [120]. Another noteworthy observation is that, in tumors with abundant autophagic stroma, the CAF signature of fibroblasts is instigated by oncocyte-derived ROS [121], which subsequently supply essential nutrients for neoplastic cells [122,123]. How to trace the reciprocity in tumor–stroma crosstalk has become a question that must be considered during model design.

#### 2.2.4. Interaction Within Stroma

The predominant form of intra-stromal interaction is ECM remodeling, driven by CAFs [124,125]. For example, Chen et al. detected macrophage M2-polarization via CXCL12 up-regulation mediated by CAFs, intensifying the malignant behavior in HCC [126]. Similarly, as Timperi et al. have reported, STAB1^+^ TREM2^high^ lipid-associated macrophage recruitment is instigated by CXCL12–CXCR4 axis activation mediated by CAF, and followed by immunosuppressive TME in breast cancer [127]. In the opposite direction, tumor-associated macrophages dominated the transition from normal breast-resident fibroblasts to CD10^+^ GPR77^+^ CAF, via CCL18 secretion and subsequent chemical resistance in breast cancer [128]. Beyond the immune system, pericytes promoted angiogenesis in lung cancer through the Wnt signal transduction obstacle and SERPINE1 expression upregulation [129]. Conversely, communications within cellular elements of narrow stroma, such as crosstalk among CAF, MSC, or pericytes, are rarely reported. One possible reason is that the trade-off between complexity and practicality in in vitro models does not support multicellular co-culture systems that could effectively demonstrate the influence of intra-stroma interactions on neoplastic cells.

### 2.3. Adaptive Dynamic Remodeling

The bidirectionality of tumor stroma remodeling pronounces this essential dynamic process in stroma-targeted therapy development, which means it can accentuate malignant bio-behaviors, including tumor invasion [130], immune escape [131], angiogenesis [132], and treatment resistance [133]. It also suppresses cancer deterioration when serving as a biological remedy [134] or as a principle anti-cancer target [131].

#### 2.3.1. Multidirectional Remodeling Process

When induced by a volatile TME, the equilibrium of remodeling is often disturbed, and adaptive stroma remodeling will confront radically different processes. For example, during tumor invasion, ECM located in a basement membrane undergoes degradation, before EMT, and the migration of a neoplastic cell [135], while inflammatory TME mediates tumor fibrosis, resulting in stroma stiffness via fiber synthesis [134], which signifies inflammatory cancer transformation in approximately 20% of malignant tumors [136], such as Hepatitis B-related HCC [137]. Additionally, Ono et al. verified that exogenous TME changes, for instance, gemcitabine treatment, up-regulate ECM related genes, including N-cadherin, TNC, and COL11A [138]. These findings highlight the intricate equilibrium and pathological imbalance in stroma reshaping, combined with multidirectional reformulating progress for TME adaptation, emphasizing the complexity in stroma-targeted modeling.

#### 2.3.2. Ambiguous Bilateral Relationship

Besides CAF, other stroma-related cells are also involved in stroma reconstruction. For instance, hypoxic pancreatic stellate cells can regulate ECM architecture via procollagen-lysine, 2-oxoglutarate 5-dioxygenase 2 overexpression [139], and MSCs participate in tumor stroma reshaping via up-regulating specific growth factors and matrix metalloproteinases (MMPs) [140]. Li et al. identified a pericyte subtype characterized by high expression of TCF21 and deactivation of the FAK/PI3K/AKT/DNMT1 axis, orchestrating increased perivascular ECM stiffness by collagen rearrangement [141]. On the other hand, the dynamic remodeling influences stroma-related cell composition, abundance and activity. As a proof, invasive margin stromal remodeling generates variation in the abundance and activity of stimulatory type 1 conventional dendritic cells, thereby eliciting cell-inflamed tumor microenvironments [142]. Consequently, the obscure causal relationships between ECM change and cell change hinder stroma remodeling replication in artificial environments.

#### 2.3.3. Cascade Bio-Effect

The bio-effects of stroma reshaping manifest at multilevel levels. In many cases, the focal point, rather than stromal remodeling itself, is the terminal of this cascade bio-effect. Chen et al. presented the immunosuppressive terminal bio-effect of hypoxia-induced stromal remodeling in scirrhous HCC, and more specifically, dendritic cells inhibition via SPP1-CD44 axis and probable T cells deactivation [143]. In another scenario, ECM stiffness regulation, achieved by collagen and proteoglycans remodeling, enhances angiogenesis in neuroblastoma, via the YAP/RUNX2/SRSF1 axis [132]. A similar phenotype can be found in colorectal cancer [97] and bladder cancer [144]. Unfortunately, an in vitro model that simultaneously contains the stroma remodeling process and end effectors, such as the immune system or vasculature as in the above studies, is such an intricate system that possesses suboptimal feasibility.

### 2.4. Multidimensional Stroma Heterogeneity

Represented by a complicated subpopulation of stromal cells [60,61], heterogeneities within tumor stroma extend across various dimensions, comprising temporality [145], spatiality [146], individuals [147], and tumor type [148]. Disparities in these perspectives underscore the challenges of personalized stroma modeling within the context of the precision medicine trend.

#### 2.4.1. Temporal Stroma Heterogeneity

Paralleled with the clonal evolution of tumor cells throughout the entire disease course [149], stroma signatures at each tumor stage receive specific modulations, induced by certain oncocyte clones and unique TME, contributing to time-related heterogeneity in tumor stroma [150]. For instance, Venning et al. employed a multicolor flow cytometry strategy to identify the CAF composition of triple-negative breast cancer samples from tumor-bearing mice in weeks 1, 2, and 3, and revealed that the abundance and dynamics of each CAF subpopulation fluctuated over time [145]. As another piece of evidence, Davidson et al. estimated three temporally specific stromal populations, conserved across mouse and human melanoma, via scRAN-seq [13]. However, due to the limited observation period compared to the clinical disease course, it remains unrealistic for most research models to manifest the whole process of stroma evolution and spot its temporal heterogeneity.

#### 2.4.2. Spatial Stroma Heterogeneity

Encompassing both inter- and intra-tumor heterogeneity [151,152], spatial heterogeneity in tumor stroma focuses on the phenotypic, transcriptomic, epigenetic, and genomic distinctions among different spatial spots within a single tumor, or between primary, satellite, and metastasis lesions [150,153]. As Wilpe et al. observed, significant differences were discerned in tumor stroma among primary sites, including lymph node metastasis and distant metastasis of urothelial cancer. Immune cell densities in lymph node metastases, including CD3^+^, CD8^+^, FoxP3^+^ and CD20^+^ cells, exceeded those in bone metastases [154]. Additionally, as demonstrated by Zhang et al., proliferative fibrosis by ECM remodeling and CAFs with obvious FOS and JUN activation was enriched in tumor regression zones mediated by immune system [155]. Thus, the importance of research concerning local reaction treated by stroma-targeted drugs is self-evident. However, the insufficient physical size and homogeneous incubation environment in most in vitro systems have dictated their disappointing potential in capturing spatial stroma heterogeneity. Stroma heterogeneities in specific patients, termed as individual heterogeneity, also emphasize modeling difficulty in precision and personalized research [156].

#### 2.4.3. Heterogeneity Among Different Tumor Types

Due to variations in tissue histologic origin and pathological processes, enormous stromal heterogeneity among assorted cancer types has been verified [150]. For example, the gene signature of CAF subtypes isolated from cutaneous squamous cell carcinoma (CSCC), including CXCL12, JUND, TSC22D3, MMP11, TAGLN, and COL11A1, shows a low fitting degree when matched with CAF subtypes from basal cell carcinoma [148]. Most current research chose patient-derived samples to preserve the features of each cancer type [157], whereas real challenges emerge in identifying the heterogeneity of certain tumors.

## 3. Preclinical Models in Stroma-Targeted Therapy Development

There is no question that preclinical stroma mimicry models possess satisfying fidelity and practicability to propel the development of stroma-targeted therapy in the precision medicine era [158]. With increasingly intensive investigations in major challenges in modeling, a diverse array of models has been deployed to faithfully recapitulate specific stromal element or dynamic process during regimen estimation. These models fall into four groups: in vivo, in vitro, ex vivo, and in silico models [159].

### 3.1. In Vivo Stromal Models

For now, even the most sophisticated in vitro models fall short of replicating the complex environment found in the simplest model organisms, such as fruit flies [160]. That is why in vivo models, considered the most classic one, persist continuously and evolve in building technologies [161] and standard specifications [162]. In spite of the notable differences in tumor stroma between rodents and mammals and the availability of more budget-friendly model animals for stroma imitation, including zebrafish [163], beagle dogs [164], pigs [165], felines [166] and primates [167], mice and rats remain the most prevalent tumor-bearing hosts, due to their genome size, which is comparable to that of humans, short reproduction cycles, large litters, low maintenance costs, and convenience in operation, which are also emphasized in this section.

#### 3.1.1. Spontaneous and Experimental Tumor Animal Models

As one of the most classic tumor models, spontaneous tumor animal models refer to naturally occurring tumors in animal models, which are commonly observed in breast cancer and leukemia models [168]. In 1915, the Japanese pathologist Katsusaburo Yamagiwa successfully achieved tumorigenesis by applying coal tar to the ears of rabbits, marking a milestone as the first artificial tumor model [169]. Since then, experimental tumor animal models, instigated by carcinogens or carcinogenic radiation, have progressively supplanted spontaneous ones in stroma-related research [161,169]. For instance, Makino et al. intraperitoneally administered diethylnitrosamine to C57BL/6/129 background mice, and the chemically induced HCC models have played a pivotal role in identifying the positive feedback loop of STAT3-CTGF in tumor–stroma crosstalk as a therapeutic target [170].

#### 3.1.2. Genetically Engineered Mice Model (GEMM)

GEMM refers to genetically modified mouse models constructed through the manipulation of mouse genes, typically involving transgenic overexpression of oncogenes or silencing of tumor suppressor genes in cancer models [171]. For instance, Kras^LSL-G12D/+^; Trp53^LSL-R172H/+^; Pdx-1-Cre (KPC) mice, utilized by Olive et al. to estimate the stroma remodeling interfering effect of the Hh pathway inhibitor IPI-926, exhibit mutant Kras and p53 in pancreatic cells, along with high carcinogenicity and human-like pathological and molecular signatures in tumor stroma [172,173,174]. The biofunction evaluation of certain genes by gene knockout in GEMM is also worth investigation. For example, the absence of MMP-9 expression in stroma-related cells in MMP-9^−/−^ mice on a C57BL/6 background decelerate the synthesis of supportive tumor stroma and relevant immunosuppression, indirectly revealing the stroma-targeted mechanism of amino-biphosphonate treatment [175].

Another factor which deserves attention is that, despite the faithful reflection of tumor progress, complications in the genome in genetic engineering approaches, as well as unrevealed antigens, can hinder the study of immunoresponses and CAR-T regimens [176].

#### 3.1.3. Xenograft Model

Xenograft models, tumor models constructed by inoculating tumor from other species into immune-deficient animals, have gained prevalence in stroma-related research [159]. The most common inoculums are assorted human-derived tumor cell lines, leading to the designation of cell line-derived xenograft models (CDX) [177,178,179], which are capable of anthropomorphic tumor–stroma crosstalk [180]. To further advance human incorporation of not only oncocytes but also stromal elements, stromal cells, like CAF or HPSC, are blended with neoplastic cells as inoculums [181,182,183]. Levental et al. employed a breast cancer organoid as an inoculum to construct organoid-derived xenograft models (ODX) for ECM estimation of type III collagen crosslink, which have been eagerly imitated by other studies [184,185].

In response to the limitations of CDX, including the excessive loss of homogenization and heterogeneity [186], that result in misaligned predictions about stroma-targeted effects [187], tumor fragments obtained from clinical specimens substitute the immortal cell line as inoculums to establish patient-derived xenograft models (PDX) [186,187,188]. Expanding beyond tumor debris, PDTOs, and even organoids generated from PDX (PDXO), can be utilized as inoculums to strike a balance between high-throughput drug screening as ex vivo preliminary experiments, and subsequently precision verification in personalized stroma [189]. These are, respectively, named as PDO-derived xenograft (PDOX) and PDXO-derived xenograft (PDXOX) [190,191].

Given the diversity in tumor origin, the xenograft model offers a variety of inoculation approaches. While subcutaneous xenografts are widely popular for their ease of transplantation and the potential for real-time monitoring [192,193], orthotopic models provide an authentic TME for stroma formation and remodeling [194]. Meanwhile, alternative methods are applied to mimic certain stroma in special TME, such as intraperitoneal injection for abdominal cavity metastasis [193,195], intra-tibial injection for osteolytic lesions [196] and intracardiac injection for circulating tumor cells [197]. A variety of options exist for tumor-bearing animals, with athymic nude, C57BL/6, NOG, and SCID mice being optimal for CDX, while NSG, NRG, SCID, and NOD/SCID mice are commonly used for patient-derived models [198]. Moreover, HIS mice can overcome immune deficiency in xenografts, and provide a stromal microenvironment equipped with a human immune system [199].

#### 3.1.4. Syngeneic Tumor Models

Given the challenging operational demands and discouraging success rate in HIS xenograft modeling [200], syngeneic tumor models, formed by inoculating immunologically sound inbred-strain mice, including C57BL/6 or BALB/c mice, and tissue-compatible mouse-derived tumor cell lines or tissues, have emerged as the preferred alternatives in the presence of significant differences in immune systems between rodents and primates [199]. Unlike immunodeficient xenograft models, syngeneic tumor models can preserve an intact immune microenvironment while minimizing immune rejection. They are particularly well-suited for studying immune infiltration, and responses induced by stroma-targeted therapies [161,199]. For instance, a syngeneic melanoma model, proposed by Horiguchi et al., was used as a platform to verify CAF-originated ANGPTL2 suppression of tumor progression through CD8^+^ T cell cross-priming and dendritic cell activation [201].

### 3.2. In Vitro Stromal Models

The primary advantage of in vitro models lies in their simplification, abstracting the essence of tumor–stroma interactions from the complex TME, which, ironically, is also a fatal flaw [159]. The model that maximizes this characteristic is none other than 2D cell culture. Unfortunately, even with the support of co-culture techniques and the Transwell system [202], 2D cultivation still cannot accurately replicate the characteristics of tumor stroma. Thus, 3D cell models have been developed. With affordable financial and temporal expenditures, the dimensionally bionic environments supplied by rolling cultures [203], bio-scaffolds [204] or microfluidic chips [205] create intra-stroma-like mechanical signatures through the physical integration of various cell types, significantly narrowing the gap between in vitro models and in vivo models.

#### 3.2.1. Two-Dimensional Culture Model

As the most representative in vitro model emerging in the early 20th century, the 2D culture model provides mechanical support for cells via rigid surfaces, such as glass or polystyrene [206]. Specific to stroma mimicry models, there are three distinct cultivation patterns to meet different experimental objectives, which are illustrated as follows: (1) Mono-culture, which refers to axenic incubation focusing on certain stromal cells [207]. For instance, Chronopoulos et al. employed 2D-cultured primary HPSC to identify that all-trans retinoic acid can suppress PSC mechanosensing by reducing endogenous force generation and cell stiffness, implying its tumor inhibition potential based on stroma remodeling disorder [208]; (2) Direct co-culture, a mixed cultivation system that allows cellular contact. For instance, PANC-1 cells and HPSC were mixed in a 1:1 ratio and seeded into 6-well plates by Liu et al. to demonstrate that a nanoparticle-based photodynamic method can modulate cancer–stroma crosstalk [178]; (3) Indirect co-culture, the co-cultivation of different cell types in the same liquid atmosphere without cellular contact, a condition achievable through propagation or through a Transwell system. In addition, Hogstrom et al. investigated CAF-mediated fulvestrant resistance in TNBC by nourishing MCF-7 cells with a CAF-conditioned medium, namely a DMEM medium that has been used to culture CAF for 48 h [209].

Generally, the 2D culture model endures, due to its low expenditure, short cycle, and impressive throughput. Yet, one drawback is that the dimension-reduced cultivation environment, which does not align with the physiological dimension, may lead to the loss of specific stromal information. Transcriptomics analyses have verified that the primitive biophysics condition provided by 2D incubation has incurred heterogeneity loss in CAF [210,211,212].

#### 3.2.2. Spheroids, Heterospheroids, and Multicellular Tumor Spheroids (MCTS)

A tumor spheroid refers to a simple-structured oncocyte spheroid originating from a primary cell or cell line which is mechanically polymerized by techniques including a ultra-low adhesion orifice plate, matrix embedding, hanging drops, spinner flasks, etc. [213].

Spheroids composed solely of malignant cells exhibit limited stroma stimulation, a fact substantiated by the intervention of stromal elements [214]. One solution is integrating one or several types of stromal cells into spheroids, termed as heterospheroid or multicellular tumor spheroids, respectively [215]. For example, the MCTS assembled by McKenna et al. consist of the PDAC cell line Panc-1, as well as PSC, which identified the indiscriminate cytotoxicity of banana lectin CAR-T cells against tumor cells and stromal cells [182]. Simultaneously, the co-culture technique emerges as another approach [214]. Keller et al. co-cultured MDA-MB-231 cell line-derived spheroids with CAFs to illustrate the reverse Warburg effect, which was induced by tumor–stroma crosstalk as a stroma-related target [216].

It is noteworthy that spheroids have remarkable expansion potential, and the synergy between spheroids and complementary techniques, such as 3D-bioprint [217], microfluidic chips [218], or bio-scaffolds [219] can generate stroma mimicry models that better capture physiological conditions [51].

#### 3.2.3. Tumor Organoids

As the physiological advancement of spheroids, organoids were reported as early as 1946, but underwent redefinition through iterated technologies [220,221]. Regarded as “organ buds in the culture dish”, the current definition of organoids signifies self-organized 3D-miniaturized organs deriving from adult stem cells (ASC), specially CSC in tumor organoids [131,221,222]. According to CSC origins, tumor organoids can be categorized into three primary groups: (1) Engineered cancer organoids (ECO), which represent normal tissue organoids generated from ASC or iPSC, which undergo cancerization mediated by cancerogens or genetic modification [222]. Driehuis et al. endeavored to employ human papilloma virus 16 (HPV16) for oral mucosa organoid infections as a simulation of virus-type HNSCC tumorigenesis [223]; (2) Pathological pathway organoids (PPO), tumor organoids based on CSC isolated from tumor tissue in animal models. They are exemplified by the PADC organoid, constructed by Schwörer et al. using KPC mice [224]; (3) Patient-derived tumor organoids (PDTO), signifying organoids which originate from CSC separated from surgery/biopsy specimens or CTCC. It is widely acknowledged as an ex vivo model, and hence minimal discussion will be presented here [225].

Compared with spheroids whose 3D structure relies on compulsory mechanical aggregation, tumor organoids experience an oncogenesis-like process programmed by an inherent genetic signature, which is paralleled with the formation of implantation recurrence lesions [226]. Consequently, tumor organoids more faithfully preserve pathological characteristics, encompassing the stroma accumulation and remodeling, as well as tumor–stroma crosstalk. It is crucial to emphasize that classic tumor organoids merely incorporate cancerous epithelial cells. Thus, stromal supplements are paramount for TME replication [227]. As compelling evidence, Schwörer et al. conducted a direct co-culture between PDAC organoids and PSCs, with the ratio of 1:8, in oxygen-rich or hypoxia atmospheres. The observation on the PSC alternations revealed that the synergistic impact of hypoxia and tumor-derived cytokines promotes the acquisition of the iCAF phenotype through HIF-dependent pathways, involving LIF and IL-1α. It is irrefutable that the research conclusion not only authenticates HIF-1α and HIF-2α as stromal targets for mini-molecule drug development, given that iCAF-related collagen depletion fosters tumor growth and diminishes the survival rate, but also provides an inspiration for CAR-T cell design, in which CAR expression is instigated by hypoxia response elements (HRE) and targeted against surface molecules expressed in CAFs, like FAP, or those essential for CAF phenotype transition like CD81 and CD63 [224].

The fidelity of organoids to stroma-related molecular signatures bestows upon them patient-like responses to innovative stroma-targeted therapies, while the transient cultivation period and affordable expenditure guarantee swift and high-throughput drug screening [228]. These merits render organoids a distinguished platform for stroma-targeted therapy research, and even a potential substitute for labor intensive models, like PDX [229].

However, tumor organoids are not a universal solution. A key limitation is the low throughput for chemical compound screening compared to 2D cell lines. This is due to the high costs of cytokine supplements and matrigels, extended organoid growth times, and a relatively high failure rate, all stemming from the model’s inherent complexity [30,230]. Moreover, the prolonged passage of PDTO introduces genetic drift, posing a significant menace to organoid quality and the spectrum of drug response. As illustrated by Choi et al., clonal evolution in late-passage HNSCC organoids is characterized by the loss of mutations in TP53, NOTCH1, and MAP3K13. This evolution, triggered by the absence of selective pressures, sheds light on the altered sensitivity of these organoids to radiation or cytotoxic reagent treatments [231].

#### 3.2.4. Models Based on Scaffolds

As an alternative to ECM, a scaffold creates a pathological environment that is rich in biophysical and biochemical cues that foster spontaneous 3D growth instead of compulsive mechanical aggregation, which accurately captures fundamental cell-ECM crosstalk [232]. Moreover, the stromal supplement embellishments in a scaffold enhances authenticity during stroma-relative bio-event stimulations [23]. For instance, Neufeld et al. designed a high-fidelity scaffold for glioblastoma, which is composed of a gelatin-fibrinogen mixture loaded with stromal cells, like astrocytes and microglia. In this scaffold, malignant cells exhibited similar proliferation curves, chemical responses, and genetic signatures compared to those of orthotopic xenografts [217]. Another illustrative example is presented by Martino et al., who employed a dental sponge incubated with type III collagen as a scaffold, highlighting the role of certain collagens in a dormant niche via DDR1/STAT1 signaling transduction [233].

Scaffolds serve not only as substitutes for the extracellular matrix (ECM), but also play pivotal roles as stromal components within therapeutic systems [234]. Recent studies reported the design of several implantable or injectable scaffolds for delivering therapeutic ingredients [235]. Obviously, accurate localization and controlled delivery avoid the systematic exposure induced by vasculature administration, dramatically curtailing systemic toxicity [236]. Simultaneously, the prolonged release achieved by affinity regulation enhances drug accumulation in lesions [237]. Meanwhile, intricate material selection and structure design can enhance cellular immunotherapy by supporting CAR-T cell survival, or stimulating specific niches that trigger inherent anti-cancer immune responses [236,238].

#### 3.2.5. Tumor-on-a-Chip (TOC)

The chip itself is regarded not as a stroma mimicry model, but rather as a platform that revolutionizes TME stimulation in an artificial environment through microfluids [239], since this device captures the essence of stroma mimicry: dynamics [2,240,241]. Specifically, in contrast to the passive infiltration in static incubation, TOC provides an internal engraving structure, a multi-cavity design connected by capillary channels, with microperfusion programmed by a digital algorithm and driven by a CNC micro-pump infusion system [242]. It is intricate, and various parameters in the liquid atmosphere receive precision regulations, including for mechanical stress, shear flow, organization interface direction, and concentration ingredients [243]. Thus, TOC proves apt for multicellular cultivation and interaction analysis. Moreover, it enables the fine-tuning of personalized stroma modeling, allowing precise observations of crucial stromal bio-events, such as angiogenesis [244], oncocyte invasion [245], and biological barrier reconstruction [246].

During drug development and screening, the tumor-on-a-chip (TOC) stands out, due to its impressive contrast between microscale sizes and minute specimen demands, and its capabilities for high-throughput and high-content detection [247]. Additionally, the microfluidic device introduces a circulatory system resembling blood or lymph vessels into the drug delivery system, rendering TOC an exceptional platform for stromal therapy estimation [244,247]. In their work, Haase et al. introduce the TS-MVN, a TOC design that is loaded with vascularized spheroids, where vessel density and barrier function fluctuation were influenced by pathophysiologically malignant effects. By evaluating the diffusivity, functional efflux, and accumulation of the fluorescent-conjugated drug after paclitaxel administration, the TS-MVN chip replicated the active transport and exclusion mechanisms triggered by endothelial cells [248]. In a parallel manner, the aforementioned L-TumorChip also demonstrated a high-throughput capability for drug-screening when assessing drug delivery and pharmacokinetics in the presence of stromal elements and microcirculation, and successfully identified the CAF-induced chemo-resistance during doxorubicin administration [218].

### 3.3. Ex Vivo Stromal Models

The relatively short history has led to controversies in defining in vitro models [249]. Represented by PDTO, the definition of ex vivo models in current reviews indicates models derived from tumor tissue but incubated under artificially in vivo or in vitro environments [250]. As the new generation model for stroma imitation, an ex vivo model adopts the experiences and technologies from an in vivo/in vitro model, and hence synchronously possesses their respective merits, which makes it a cost-effective option that bridges the gap between in vivo and in vitro models [159].

#### Tumor Slice Culture (TSC)

Apart from PDTO, which receives comprehensive discussion in Section 4 and Section 5, another representative ex vivo model is TSC, which is also named as patient-derived explant (PDE). The concept of TSC was pioneeringly proposed by Dr. Harford in the 1950s, and underwent refinement and applications in pharmacological and toxicological evaluation over subsequent decades [251]. During the preparation of organotypic TSC, tumor specimens are promptly incised into morphologically regular slices with an accurate thickness range from 150 to 500 μm after surgery or biopsy, followed by an ex vivo incubation on cell-culture inserts or gas–liquid interface conditions [252,253].

The predominant merit of TSC lies in its holistic fidelity, a trait validated across a spectrum of primary tumors, spanning breast adenocarcinoma, colon carcinoma, melanoma, glioblastoma, prostate cancer, PDAC, TNBC, and CRC [254,255,256,257,258]. Simultaneously possessing integral ingredients incorporating neoplastic cells, stromal elements, the circulatory system, and immune components, TSC can guarantee not only survival but also functionality, for days and even weeks. This resilience has been demonstrated in various aspects, including cytokine paracrine, vasoconstriction, calcium currents, and intercellular contact [251,259,260]. Thus, the complexity between tumor and stroma earn an authentic retention. Distinctively, TSC preserves the primitive coordinates of various constituents in the tumor, eschewing artificial or automatic re-aggregation following digestion dispersion. This strength undoubtedly lays the foundation for delving into the adjacent tumor–stroma interplay, based on cellular compartments or special niches. As evidenced by Misra et al., TSC derived from PDAC remained morphologically stable, and an analysis of cellular markers revealed that the stroma associated with either the cancer or residual pancreatic parenchyma maintained its characteristic heterogeneity [261].

Its biomimetic performances position TSC as a reliable assessment platform for cytotoxic drugs [262,263], immunotherapy [264,265], radiotherapy [253,266], and adoptive cellular therapy [256]. The efficiency of drug evaluation is further exaggerated by the yield that a single cut can fabricate, of nearly a hundred slices [257]. In the drug-screening research conducted by Zhou et al., malignant breast tumors were prepared as TSC to assess different drug combinations predicted by a quartet-synergy metric machine learning algorithm, including cepharanthine + vincristine, docetaxel + tamoxifen, lapatinib + Pazopanib, and ABT-199 + vincristine [267]. Additionally, in a similar design conducted by Martin et al., an EvG stain and a Halo platform were employed to estimate the stroma area alternation in TSC after first-line drug administrations for CRC, including oxaliplatin, cetuximab and pembrolizumab. Consequently, the research illustrated that TSC manifested high morphological comparability with the original tumor as well as realistic preservation of tumor–stroma interaction, and simultaneously declared the vital role of oxaliplatin in stroma-rich cases [268].

### 3.4. In Silico Stromal Models

The Industry 4.0 wave has propelled the evolution of in silico models, and the iterative advancements in sequencing technology have significantly enhanced the potential of in silico models for stroma mimicry [269,270]. As computational experiment models reliant on computers and servers rather than traditional bio-laboratories, in silico models have not only introduced innovative concepts for stroma simulation, but have also furnished distinctive insights into predicting stromal-targeted therapies [271].

#### 3.4.1. Public Sequencing Data

The paramount model for in silico stroma replication undeniably revolves around sequencing datasets, particularly high-resolution data about single cells and the spatial transcriptome [159]. The ubiquity of large-scale omics, coupled with the optimized sequencing technique and the ethical mandate for data publication, has exponentially increased the accessibility of public databases, obviating the need for wet-lab processes [272]. The impressive cost-effectiveness and dependable repeatability of public sequencing datasets not only reduce the rigorous technical demands for beginners, but also establish them as an endurable and shared data source.

Beyond cost containment, pan-cancer analyses rooted in public datasets lay bare stromal commonalities across diverse solid tumors, thereby expediting the identification of universal stromal targets [273]. Rohatgi et al. conducted a meticulous tumor transcriptome deconvolution, culminating in the creation of a pan-cancer metabolic atlas of the tumor microenvironment that encompassed 20 solid tumor types. While metabolically conserved signatures were unequivocally confirmed in tumor-infiltrating stromal cells, the research results also unveiled a general up-regulation of the rate-limiting enzymes for tryptophan catabolism, IDO1 and TDO2, in the stroma, which implied that kynurenine-mediated immunosuppression may be predominantly restrained by the stroma. Furthermore, stromal cells exhibited a Warburg-like phenotype in this in silico model, providing robust evidence in support of the reverse Warburg hypothesis [274]. Moving beyond a pan-cancer perspective, the nuanced longitudinal remodeling for stroma in certain tumor types, based on massive datasets, have ignited a paradigm shift towards stroma-targeted therapy.

#### 3.4.2. Machine Learning (ML)

Embedded within artificial intelligence, machine learning algorithms stand as powerful tools for predictive analysis via data pattern recognition, propelling the advent of digital pathology [275]. A prominent case of use involves prognostic prediction and pathological classification, based on a convolutional neural network trained with whole slide images, as the emergence of modern graphic processing units (GPU) can support the implementation of intricate architectures at scale [276]. For instance, Micke et al. employed an objective machine learning method to qualify tumor stroma across 16 distinct solid cancer types, encompassing 2732 patients. Their method utilized pan-cytokeratin staining and autofluorescence patterns to segment stromal and epithelial compartments, revealing substantial variances in the tumor stroma fraction among different solid cancer types. In another application, ML also provide an opportunity for efficient drug repurposing research and precise prediction of therapeutic responses, since strides forward in deep learning usher in fresh perspectives on the intricate interplay between physicochemical properties and phenotypic changes [277]. Obviously, multi-perspective exercise materials, including electronic health records, sequencing data, and public compound libraries, benefit the methodical integration of computational strategies across a spectrum of pharmacological signatures, spanning drug–target interaction, molecular docking, molecular dynamic simulation, ligand reconstruction, proteochemometrics, and cellular phenotype recapitulation [267,278]. Besides the quartet-synergy metric machine learning algorithm for drug combination design mentioned in Section “Tumor Slice Culture (TSC)” [251], the proposal of scDEAL, a deep transfer learning method for cancer drug responses, stands out as an archetype. Trained on a substantial volume of cell-line data, scDEAL manifested robustness and reliability in the benchmark examination on six drug-treated scRNA-seq datasets that were equipped with guaranteed drug-response labels. It is distinctive that, during the integration of large-scale bulk cell-line data conducted by Chen et al., drug-related bulk RNA-seq data were harmonized with scRNA-seq data, facilitating the formation of integrated gradient feature interpretation to locate the pivotally chemo-resistance genes [279].

#### 3.4.3. Computational Models

Apart from the mature paradigms exhibited above, a myriad of computational models have saturated the realm of in silico stroma recapitulation, whose logical mechanisms are so different that categorizing and enumerating them becomes a formidable challenge [280]. As a proof, the Euler–Maruyama method has been identified by Chen et al. as a potent formulation for a large system of stochastic differential equations which mathematically describe cellular displacement in the early stages of PDAC. The rational simplification of the chaotic TME highlighted the virtual stromal response after administration of PEGylated PEGPH20 in combination with gemcitabine, which was silicification though Green’s fundamental solutions of the reaction–diffusion equation. Remarkably, enhancements in T lymphocytes penetration induced by enzyme-mediated ECM degradation were precisely forecasted via calculation, which aligned closely with the realistic clinical records that the correlation coefficient between initial and terminal fractions reached 0.8785. As a supplementary arithmetic, Monte Carlo simulations facilitated the investigation for uncertainties of input parameters and anticipated recovery probability, corresponding with diverse diagnosis stages [281]. Research studies with a mechanistically consistent approach have delved into the dynamics of pancreatic cancer metastasis [282] and classic cellular immunity inhibition within tumor islets, triggered by ECM remodeling [283].

Indeed, the inherent limitation of untethering reality is inevitable when employing a computational model, but their contribution to bulk prediction with minimal expenditure also demands acknowledgment. As the ultimate embodiment of the “replace” principle within the 3R framework [284], in silico models wield the capability to optimize about drug therapies and achieve pre-validation before pre- or formal clinical trials.

## 4. Co-Culture PDTO Application in Stroma Imitation

The currently prevalent preclinical models are 2D cell lines for preliminary drug screening, and tumor-bearing animals (typically mice) for in vivo verification [213], garnering endorsement under the Federal Food, Drug, and Cosmetic Act of 1938. However, this amalgamation displays a demoralizing inadequacy in the realm of stroma mimicry, since deviations in stroma formation, accumulation, remodeling, and pertinent responses caused by the 2D cultivation environment and rodent biology culminate in discernible dissonance between the developmental and application stages in terms of efficacy and toxicity [228]. Meanwhile, the paucity of iterative trials grounded in biodiversity neglects stromal heterogeneity among individuals, eventually engendering diminished clinical response rates concerning novel therapeutics, as homogenization has been achieved in cell lines during prolonged passages, and inbreeding also causes approximately 98.6% genome sharing in the same mouth strain [285]. Thus, after a 25-year tenure, the National Cancer Institute (NCI) officially declared the overhaul and suspension of the NCI-60 panel in 2016, symbolizing the withdraw of cell lines from the erstwhile preeminent position in the domain of drug screening [286]. Another seismic news event transpired with the enactment of FDA Modernization Act 2.0, legitimizing versatile alternatives to animal testing, for example, organoids and organs-on-chips (OoCs) [287]. It is self-evident that the vacuum formed by retirement of two cornerstone models appeals the engagement of a next-generation stroma mimicry model. The comparison in Table 1 stresses the promise of ex vivo models as the competent successors to 2D cell lines for initial drug infiltration, and the surrogates of labor-intensive in vivo models.

As an embodiment of organoid in ex vivo milieu, PDTO is concretized through the 3D self-organization of tumor tissue-derived cells centered around CSC, which comply with the lineage commitment of cell differentiation and spatial constraints [288]. One merit lies in preservation of a genetic signature in the primary tumor, making PDTO a promising model that can cater to the precise medicinal wave. Another merit is that PDTO furnishes an optimal environment, which is conducive not to static mutualistic symbiosis, but to the dynamic interplay of non-neoplastic cells, enhancing its applicability for the exploration of the multifaceted influence exerted by various stromal cells on tumor progression, metastasis, and drug resistance [289]. Diverging from ECO or PPO, the primary PDTO, an ex vivo model, encapsulates a broad spectrum of cells sourced from tumor tissues, comprising oncocyte (especially CSC), immunocyte, endothelial cells from vasculature, and various stromal cells. Nevertheless, the Hayflick limit and passage operation contribute to the gradual attrition of stroma components in subsequent generation, eventually leading to the predominance of epithelial parts, like cancer cells. Hence, in pursuit of a sustained and robust recapitulation of the stroma, the supplementation of exogenous stroma via the co-culture process is imperative [289,290]. As the term conveys, co-culture involves the simultaneous cultivation of distinctive cellular types within a single environment [25]. Once induced through the co-culture, stromal elements intricately engage in intricate crosstalk with cancer cells via direct cellular contact and/or signaling factors, and cytokine secretion [30], which ensures a biologically faithful response when encountering stroma-related treatment. The strategic incorporation of exogenous stroma, characterized by its deliberate cultivation alongside cancer cells, serves as a pivotal strategy to counteract the inherent limitations posed by the telomere constraint and continued passages.

**Figure 3:**
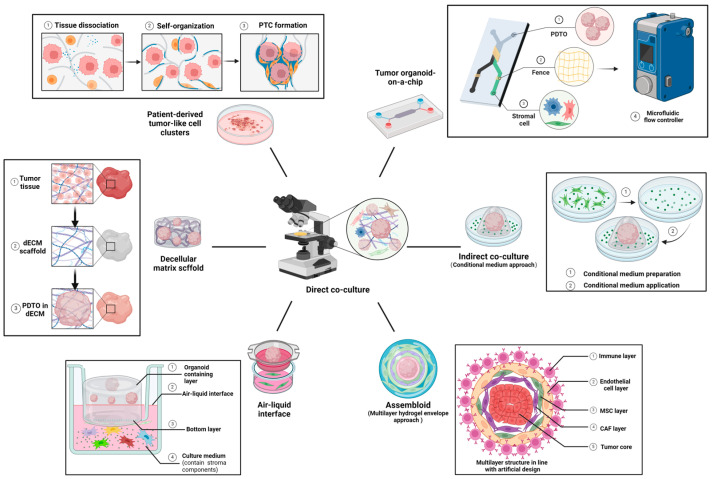
Review of different co-cultured PDTO approaches with characteristics emphasized for each model.

In this part, as shown in Figure 3, an exhaustive research illustration is performed to demonstrate various co-culture combinations and pioneering techniques in co-cultured PDTO during tumor stroma imitation.

### 4.1. Classic Co-Culture

Currently, the most prevalent co-culture paradigms can be conventionally classified into two distinct types based on their permissibility of direct contact between different cell types: (1) Direct co-culture indicates incubation that allows both spatial juxtaposition and paracrine signal transduction between different cell types, whose presentation form is usually a bulk of culture [291]; (2) Indirect co-culture indicates incubation that allows only paracrine signal transduction, without direct cellular contact. One approach is the Transwell system, which is a polyester mesh scaffold inserted into a culture dish that segregates different cells into an upper layer (cultivated in a scaffold) and a lower layer (cultivated in a dish), thereby establishing a permeable barrier that facilitates the shared liquid environment among diverse cell types [292]. Another primitive measure is the unidirectional culturing of different cell types in a temporally sequential manner, using the culture medium as an intermediary for paracrine signal transduction [293].

#### 4.1.1. Co-Culture with CAFs

As the most prominent elements in tumor stroma, CAFs have received extensive attention for co-culture with PDTOs derived from assorted solid tumors, including breast cancer [209], gastric cancer [294], OSCC [295], HCC [296], PDAC [297], CRC [190], etc. There usually exists a relationship of mutual interplay between CAF and tumor organoids; the unique niche provided by this biomimetic model determines the phenotype and subtype of CAF, which reciprocally regulate the biofunctions related to neoplastic cells, involving matrix remodeling and subsequent tumor invasion and metastasis, tumor metabolic reprogramming, as well as radio-and chemo-resistance. For example, through co-culture with a PDAC organoid, Schwörer and his colleagues reported the phenotype transition of CAF from myofibroblastic (myCAF) to inflammatory (iCAF) status, which was instigated by the fluctuation of HIF-1α signaling, and accelerated by cancer cell-derived cytokines, like IL-1 and TNF-α [224]. However, under a CRC background, a converse conclusion was drawn by Mosa et al. since co-culture with PDTO significantly enhanced myCAF differentiation together, characterized by Axin2 expression [298]. From a reversed perspective, the CRC organoid and CAF co-culture experiment conducted by Mosa indicated that the fluctuation of classic Wnt signaling in CAFs led to distinct phenotypes, where decreasing and increasing levels, respectively, induced an iCAF, or a contractile myCAFs subtype. In contrast with PDAC, where αSMA^+^ myCAFs constricted tumor progress [299], a trophic function was detected in the myCAF enrichment niche. Moreover, co-culture with iCAFs facilitated the EMT process of CRC organoid via IL-1 and BAFF pathways, while myCAFs reverted this phenotype [298].

The majority of CAF-dominant reciprocal crosstalk magnifies malignant bio-behavior in tumors, whose clinical manifestation is usually drug-unresponsiveness and a poor prognosis. As a valid proof, Liu et al. elucidated that CAFs established a stromal niche, offering trophic effects for liver cancer PDTO via cytokine paracrine involving the HGF pathway. Similarly, Zhao et al. designed a direct co-culture system to identify lactic acid as CAF-induced stemness augmented in the OSCC organoid, which was characterized by increased expression in CD44 and OCT-4 [295]. Closer to clinical treatment, a high take-rate protocol for the synchronous propagation of PDTOs and matched CAFs from primary and metastatic HR^+^ breast cancers was formulated by Hogstrom and colleagues, in which there existed a mixture of different subtypes CAFs, depending on the different pathological phase and original location of the tumor specimen. Impressively, the mono-culture PDTOs derived from the recurrent case exhibited a confusing sensitivity to fulvestrants, while also manifesting a drastic response when in CAF-conditioned media. Further exploration based on a cytokine array indicated IL-8 as the cytokine with the maximum yield, but GROα and CCL19 as the chemo-resistance-relevant factors. Meanwhile, RNA-seq analysis revealed an upregulation trend in cytokine-responsive pathways among indirect co-culture PDTOs, multiple of which, like the ER pathway, can induce resistance to fulvestrants [209]. All these research studies remind us that stroma-mediated chemo tolerance can be a joint target when performing cytotoxic drug administration, and the novel strike approach may rely on the recruitment of tumor-suppressive subtype CAFs, as well as the manual interposition of adverse phenotype transition.

The kernel of personalized therapy guided by the precise medicine principle lies in the appreciation of individual heterogeneity, which has partially reappeared in various PDTO bio-banks [191,300]. Imperfectly, the current attention is conspicuously attached to neoplastic cells, while the contribution of stroma elements, like CAF, to these divergences has been neglected. As a result, the homogeneous incubation condition effaces many distinctive pathological features, such as factitious bias, in consensus molecular subtypes (CMS). In order to record the individual heterogeneity lying in not the tumor itself but in stroma, Farin et al. established an organoid–stroma bio-profile, comprising 30 matched pairs of CRC PDTOs and syngeneic CAFs originating from a single patient [190]. With the presence of CAFs, the defection of certain “essential growth factors”, like Noggin and EGF, did not interrupt tumor-inherent signaling transduction, but were impressively reprogrammed based on the pathological features of different CMS, which supported the conclusion that a less instructive incubation condition contributed to a more informative model. Additionally, the author team postulated that the intervention of certain stroma elements intimating with drastic chemo-resistance or a disappointing prognosis could generate more predictive models for therapeutic strategy. As a proof of this clinical intervention assumption, CMS-specific drug responses were completely eliminated in monocultures under standard incubation conditions. When utilizing co-culture bio-files, a dominant restriction in the gefitinib effect was detected in the CMS4 subtype, since CAF-secreted cytokines, including TGF-β, HGF, and FGF family ligands, that can serve as compensation for EGFR-signaling truncation [301]. Consequently, organoid–CAF co-cultures are instructive in the realization of the entire capability of PDTOs for personalized oncology.

#### 4.1.2. Co-Culture with Other Stromal Cells

Stellate cells are commonly found at, heightened concentrations and exhibit activity primarily within pancreatic or hepatic tumors. Beyond their role as CAF progenitors, stellate cells contribute significantly to diverse stroma remodeling processes through the secretion of ECM-related elements. This is exemplified by the pronounced desmoplasia observed in PDAC, or the induction of fibrogenesis and carcinogenesis in para-cancerous liver tissue. Currently, the bidirectional influences exerted by stellate cells have been substantiated, underscoring their enigmatic role in the promotion, progression, and metastasis of cancer [87,302]. A research study conducted by Yan et al. reported statistically significant ERK 1/2 phosphorylation in activated stellate cells, which dominated not only cellular senescence and autophagy, but also enhanced invasiveness and viability via the EMT process in a co-cultured PDAC organoid [303]. Furthermore, Hahn et al. established human PDAC organoids from surgical samples, and co-cultured them with a stellate cell to assess the tumor-suppressive effects of metformin, a first-line drug for type 2 diabetes. Interestingly, TIMP2 signal and consequent invasive states in the PDAC organoid was activated by MMP2 expression in stellate cells, since the PDAC organoid itself rarely displayed MMP2 expression. Thus, as a stroma-related target, MMP2 downregulation in stellate cells after metformin administration accounted for the reduction in TGF-β-related downstream-signaling molecules, while the recombinant TGF-β supplement reversed the metformin-induced EMT phenotype and anti-migration effect caused by MMP2 fluctuation [41]. All these studies underscore the significance of considering the stromal environment in the evaluation of repurposing existing drugs, as its absence may lead to inconsistent false-negative results with actual applications.

The primary functionalities ascribed to MSC are inherently linked to repair mechanisms and regenerative activities, persisting even within tumors, which have been metaphorically depicted as “a perpetual wound that never heals” [6]. In the realm of tumor–stroma crosstalk, MSCs assume a pivotal role in certain TME occasions, particularly through cellular fusion with neighboring tumor-associated cell populations. This dynamic interaction engenders MSC-mediated features that can either inhibit or promote tumor progression, contingent upon the nuanced interplay of factors, such as the availability, concentration, and synergistic effects of stimulating factors within the localized microenvironment surrounding MSCs [304]. As Dhimolea et al. reported, the comparison between mono-culture HR^+^ breast or prostate cancer PDTO and co-cultured PDTO with BMSC identified BMSC-induced hormone-independent proliferation, and the restoration of the lumen filling, as the stroma-relevant resistance mechanism of histone therapy (HT). Furthermore, the presence of BMSC achieved paracrine IL-6-induced HT tolerance via the attenuation of HR protein expression, and IL-6-independent resistance via the acquisition of redundant compensatory signals, ensuring anchorage independence via ERK and PI3K bypass cascades [305]. However, through reverse thinking, Eliopoulos et al. retrovirally engineered BMSC for the extension of the IL-12 secretion, and even established it as a neo-organoid. The co-culture result with breast cancer cells indicated a substantial interference in tumor growth, accompanied with an enhancement of IL-12 and IFN-γ yields. The syngeneic mice models receiving co-injection of the modified BMSC organoid and tumor cells possessed less tumor lesion, necrotic tumor islets, and necrotic capillaries, implying that anti-angiogenesis is a potential mechanism, beyond oncocyte-killing [306]. These “opposite” conclusions uniformly inspire us that MSC can serve both as a fragile link in the crosstalk chain and as a mercenary through an artificial program.

Presently, an advanced strategy for simulating tumor stroma based on organoids is undergoing continuous refinement, achieving validations across diverse solid tumors. It is notable that the protagonist of organoids is not a tumor, but normal or pre-cancerous tissue organs, and the subsequent invasion or inoculation of cell lines, primary neoplastic cells, or even tumor organoids is capable of triggering the pathologically stromal features, abiding by the intrinsic tumorigenesis mechanism. The subsequent invasion or inoculation of primary neoplastic cells or cell lines is demonstrated to induce pathologically relevant stromal features, aligning with intrinsic tumorigenesis mechanisms. For example, Mertz et al. designed an adipocyte- or preadipocyte-encapsulating geometrically inverted mammary organoid, whose adipose core was enveloped by a cell-produced basement membrane. Through the seeding of TNBC cell lines, like MDA-MB-231, the dynamic invasion process across the epithelium layer and through into the adipose core was faithfully observable by virtue of the confocal image stacks of whole mount specimens. Furthermore, a collagen synthesis and accumulation in the cancer-associated connective tissue was detected, representing ECM remodeling conducive to invasiveness. The valid evidence for this was that the tumor invasion manifested an impressive sensitivity to anti-fibrotic drug pirfenidone [307]. Indeed, this approach demonstrates a nuanced appreciation of the intricate nature of tumor stroma, prioritizing the faithful reconstruction of its complexity, rather than exclusively centering on tumor cells. As stroma-related targets ascend from peripheral roles to central prominence, this platform could potentially serve as an enhanced foundation for drug development.

#### 4.1.3. Co-Culture with Other TME Components

The exploration of biomimetic vascularization in patient-derived tumor organoids (PDTOs) represents an enduring frontier in research. The lack of capillary-mediated microcirculation not only distorts nutrient-exchange patterns and omits hydromechanical properties, but also impedes the crucial paracrine communication between tumors and endothelial cells. For instance, Choi et al. described a HUVEC-PDAC co-cultured PDTO with a vascular niche, providing an optimum TME for CD24^+^CD44^+^ cancer-initiating cells, as an alternative for the matrigel matrix with stem cell niche factors. In a detailed manner, endothelial cell-derived cytokines achieved Wnt activation through the Notch independent pathway. However, sprouting and angiogenesis were not observed in this composite model, which can be explained by the lack of crosstalk associated with macrophages, CAFs, and pericytes [308]. In a similar research study conducted by Lim et al., a protocol conducive to the co-encapsulation of HCC-PDXO with endothelial cells through a 3D hyaluronic acid hydrogel matrix, instead of biologically ambiguous preparation, like Matrigel, was drafted to evade the background noise in angiocrine crosstalk exploration. In contrast with the mono-culture of organoid or endothelial cells, the conspicuous upregulations of MCP-1 and IL-8 expression in HUVEC, and TNF signal-related molecular expression in oncocytes, were detected as straightforward support for establishment of cellular contact-dependent angiocrine crosstalk. Moreover, the enhancement of chemokine secretion and macrophage polarization indicated that endothelial cells stimulate the inflammatory microenvironment in HCC through the recruitment of immune cells [309].

The research purpose represented by the immunological PDTO co-culture can be summarized in three types: (1) Immune microenvironment recapitulation. For example, Zhou et al.’s work proposed a conception for efficient drug-assay array, based on the co-culture of PDAC organoid and T cells. A two-step cell packaging approach was employed to envelop tumor-specific T cells in the frontier region of the PDTO, in line with the inconspicuous immune infiltration and stroma barriers in tumor tissue. Furthermore, F4/80^+^CD11b^+^ tumor-associated macrophages (TAMs) and Treg cells in primary PDTOs were faithfully preserved in biomimetic proportions, as in original tumors. Consequently, the detection of T cells exhaustion verified that the complex organoid platform reappeared in a physiologically immunosuppressive environment that photocopied TME in pancreatic cancer, illustrating the value of a combined therapeutic strategy involving immune recovery [310]; (2) Antitumor immunity assessment. Through a co-culture system incorporating cholangiocarcinoma (CCA), PDTO, and peripheral blood mononuclear cells (PBMCs)/effector T cells, Zhou et al. observed a fluctuating, variable cytotoxic effect in organoids via anti-tumor organoid immune responses. In the PBMC group, the proportion of CD4^+^ and CD8^+^ T cells significantly exceeded that of NK cells, indicating more efficient immune killing in the T cell enrich group. Meanwhile, excessive IL-2 magnified the organoid death that was characterized by an increase in CYFRA, demonstrating an immune plasticity in this artificial system [311]; (3) Generation of therapeutic cells. As Dijkstra et al. reported, co-culturing with autologous CRC or NSCLC PDTOs could assist the enrichment of tumor-reactive T cells from peripheral blood lymphocytes, which was regarded as an unbiased method for the recruitment and isolation of tumor-reactive T cells, optimum for assessment of the cyto-damage efficiency to matched tumor organoids [50].

The upsurge in research on tumor microflora, a newcomer of TME, stimulates the co-culture experimentation between tumor organoid and microbes. In a bold attempt, Gao et al. utilized the CRC organoid model to investigate the influence of *Fusobacterium nucleatum* on the PD-L1 blockade. After the introduction of *F. nucleatum,* malignant proliferation was dramatically restricted in the organoids by anti-PD-L1 mAb, implying that exposure to certain microbes augments the sensitivity of clinical CRC samples to PD-L1-related therapy. Further exploration demonstrated that the upregulation of cGAS, and activation of NF-κB signaling by phosphorylation of p65, may serve as a potential molecular mechanism [312]. Parallel research focusing on *Helicobacter pylori* was also conducted by Holokai et al. in the context of gastric cancer, whose result supported the finding that *H. pylori* with excessive PD-L1 expression may lead to immune escape, incurring premalignant lesions progressing to gastric cancer [313]. Consequently, a hypothesis become gradually clear: Can genetically modified pathogenic bacteria serve as an auxiliary to immunotherapy?

### 4.2. Next Generation Co-Culture

The accumulated research has revealed that, during the development of a co-culture PDTO system, the simplistic application of matrigel to co-embed tumor and stromal components or their placement together within a singular liquid environment may prove inadequate for generating organ-like structures with incorporated stromal cells [25]. Furthermore, the primitive equipment utilized in typical co-culture scenarios falls short in reproducing sophisticated stromal properties, such as fluidic dynamics. Consequently, various tissue engineering technologies are utilized to orchestrate co-cultures endowed with heightened physiological characteristics [314].

#### 4.2.1. Tumor-Organoid-on-a-Chip

Microfluidic platforms, typically presented by chips, are devices equipped with micron order channels and chambers, which have been extensively utilized in organoid co-culture for stroma mimicry. The intricate design of their interior structure endows the chips with a precise manipulation capability of a public medium in a co-culture system, such as micro-fluids or a matrix, enabling a controllable environment for TME properties, including hypoxia, nutritional gradient, acidity, and even vascular dysfunction [315].

A multi-layer microfluidic chip, loaded with hepatocellular carcinoma (HCC) organoids, was designed for high-content co-culture and high-throughput drug screening. In addition to patient-derived tumor organoids (PDTOs), the system incorporated peripheral blood mononuclear cells (PBMCs), mesenchymal stem cells (MSCs), and cancer-associated fibroblasts (CAFs), enabling synchronized co-incubation with an acceptable cellular resolution. This setup effectively demonstrated tumor–stroma interactions, where PDTO growth was significantly accelerated by CAFs or MSCs, reducing the establishment time from 4 weeks to under 2 weeks. Stromal cells also induced the recruitment and activation of monocytes into M2-type tumor-associated macrophages (TAMs), despite the challenges associated with prolonged culture conditions. In the drug screening phase, the MSC-PDO-PBMC microfluidic chip exhibited a more predictable response to PD-L1-targeted therapies, such as Atezolizumab, faithfully recapitulating the tumor microenvironment and immune interactions. The chip design featured tailored microarray units, which were one-thousandth the size of wells in a 96-well plate, providing optimal control over dimensions and drug delivery, making it both cost-effective and practical for commercial drug evaluation [227]. In another important research study about organoid chip utilization in stroma imitation, conducted by Haque et al., a tumor chip comprising PDAC organoids, pancreatic stellate cells, and macrophages was manufactured as a promising platform for stroma simulation. Impressively, the microfluidic device loaded with assorted stromal cells not only extended cellular biofunction and longevity in PDTOs, but pathologically recreated the dynamic organotypic environment, characterized by desmoplastic stroma. Moreover, the adjuvant therapy, using stroma-depleting agents, like all-trans retinoic acid or liposomal clodronate, obviously exaggerated the effect of chemotherapy on PDTO, validating this tumor chip device as an effective tool for the development of a stroma-targeted strategy [316]. However, it should be acknowledged that several deficiencies disturb the further application of organoid chips in stroma investigation, including the adsorption of chip material to paracrine cytokines, and the limited space for organoid expansion [242].

#### 4.2.2. Assembloids

The spatial rearrangement between different elements in a typical PDTO co-culture system usually relies on a self-organization mechanism, and leads to a disappointing bulk of culture with an unsatisfied cell resolution. A shortcut to omit biological reshaping is artificial assembling, according to an inherent structure, through tissue engineering techniques, for instance a multilayer 3D-bio-print or hydrogel encapsulation, which guarantee the adjacent crosstalk in a not spatially chaotic, but pathological manner [317]. In an authoritative case study conducted by Kim et al., bladder tumor assembloids were generated through the successive co-culture of PDTO, CAFs, the HULEC muscular layer, and even the CD8^+^ T cell in a rotary cell culture system for weeks. After bio-integration, morphological changes were reported, accompanied by tumor growth in reconstituted assembloids, which eventually align with phenotypes in parental tumor tissues. It deserves attention that the intervention of CAF terminated the spontaneous subtype’s switch from luminal to basal in continually cultured PDTO, emphasizing the stromal signal role in phenotype activation and preservation. By virtue of the definite macrostructure, different infiltrating states from the stroma compartment to the muscle layer were identified. Other responses initiated by tumor–stroma crosstalk included the formation and connection of the capillary network, promoting nutrient delivery and tumor growth. During drug response assay, assembloids recapitulated their obvious tolerance to conventional chemotherapy drugs, as well as their reliable response to stroma-mediated, subtype-specific anti-cancer treatments, which can be demonstrated by limited delivery, because of the surrounding stroma. Moreover, the high-throughput 3D bio-print version manifested a potential for stroma-related drug evaluation [318]. In a separate study, Langer et al. presented a versatile assembloid design for solid tumors, utilizing 3D bioprinting. This protocol, validated using breast cancer and pancreatic cancer models, enables the precise integration of extensive tumor tissue in a defined spatial configuration, incorporating both cancerous and stromal cell populations. During the assembly’s maturation process, significant phenotypic changes were observed, particularly in relation to stromal components. These included the deposition of the extracellular matrix (ECM), the reorganization of endothelial cells into adaptive networks, and the directional migration of stromal cells, resembling the stromal fluid dynamics seen in desmoplastic tumors. Notably, the stromal composition can be systematically modulated by adjusting the proportions of various stromal cell types, such as adipocytes secreting leptin or mesenchymal stem cells (MSCs), that enhance collagen accumulation and maturation [319].

#### 4.2.3. Bionic Scaffold

The prevalence of commercial standard scaffolds, like Matrigel, and emerging ultra-low attachment consumables in classic PDTO mono- or co-culture setups must be acknowledged. However, this prevalence results in the oversight of multiple phenotypes and intrinsic adaptations in the ECM, involving inflammation, hypoxia, fibrosis, and connective tissue proliferation [320]. As a relatively convenient solution, decellularization is applied to remove cells while preserving collagen structure, ecological niche, and the fundamental characteristics of the tissue’s origin, such as stiffness and presence of desmoplasia [321]. For instance, Tienderen et al. prepared dECM derived from liver tissue as a physiological scaffold (CCAM), and dECM derived from tumor tissue as pathological scaffold (TFL-M), for cholangiocarcinoma PDTO incubation. As suggested by scRNA-seq analysis, the transcriptomic profile of PDTO received a fidelity improvement in the CCA environment when compared with tumor organoids in the TFL-M or in the standard conditions. From a clinical perspective, CCAM upregulated chemo-resistance to the first-line drug combination, gemcitabine with cisplatin. It is attractive that, as an invasion symbol, the collective oncocyte migration happened within TFL-M, characterized by an overexpression of ITGB2-AS1 and an invasive front phenotype. However, singular migration was detected within the CCAM, with gene set enrichment focusing on EMT. A further exploration unveiled that a desmoplastic environment could trigger the synthesis and accumulation of ECM components (COL1A1, COL3A1, FN1), even with the absence of CAFs, offering inspiration for exploring matrix-targeted therapies affecting the fibrous environment [322]. A similar research that also focuses on the CCA was conducted by same team. However, dECMs from lymph nodes and lung tissue were prepared as a metastatic niche. When cultivating PDTO in decellularized tissue, metastasis-relevant properties, including EMT and CSC plasticity, were intentionally altered by the ECM in an organ-specific manner, represented by divergences in migration and the proliferation dynamics [323].

Gel-like and solid structures are not inherent characteristics of scaffolds. Shan et al.’s recent work utilized a vortex acoustic field to construct an acoustic virtual 3D scaffold (AV-Scaf). This channel, with its ability to stimulate calcium ion channels, promotes organoid self-assembly and interactions between heterologous cells. Compared to the matrix gel, AV-Scaf emphasizes the enhanced cytotoxicity of T cells against tumors under co-culture conditions, with a significant upregulation of granzyme B (GZMB) and interferon-γ (IFN-γ) secretion [324].

Nevertheless, the uncritical acceptance of natural materials without thorough analysis and comprehension of their bio-ingredients does not allow for the isolation and investigation of singular features, creating problems in tracking the physical/chemical cues that are essential in niche formation, and hindering the exploration of molecular mechanisms in tumor–stroma crosstalk.

#### 4.2.4. Air–Liquid Interface (ALI)

There are various tumor types, such as respiratory tumors, like NSCLC, and tumors occurring in the skin or oral cavity, like HNSCC or melanoma, which can directly interact with the external environment. This renders traditional culture conditions, involving complete embedding in gel or submersion in liquid, insufficient to accurately mimic the physical environment of tumor growth. Using a permeable membrane at the bottom of suspended chambers, ALI technology allows one side of the culture to be in contact with a liquid medium while the other side is surrounded by air. Therefore, the air-liquid interface (ALI) technique is well-suited for studying tumor tissues that are directly exposed to the atmosphere, aiding researchers in more accurately simulating in vivo environments [325]. Neal et al. adapted the air–liquid interface technique that had been employed in the development of normal tissue organoids, such as skin and airways [326], to integrate PDTO originating from melanoma, NSCLC, or RCC with immune and stromal elements. Crucially, a tumor-like architecture and stroma marked by SMA and Vimentin were found in PDTO, which were evidence of the presence of myCAF. Moreover, with the assistance of ALI, distinctive growth patterns, as well as stroma heterogeneity, were detected in PDTO, depending on the pathological grade and initial condition of biopsy. Similar divergences were also observed in TIL activation, expansion, and cytotoxicity responses, attributed to inherent differences in tumor and immune composition, as well as resistance to checkpoint inhibition. It is noteworthy that endogenous immune cell types, including macrophages, T, B, and NK cells were consistently preserved instead of late supplementation or reconstitution through clonally expanded or TCR-engineered TIL populations. For instance, supported by a scRNA-seq assay, PDTOs from diverse donors restored all main immune lineages. This conclusively illustrated that TILs in PDTO faithfully preserved the TRC spectrum in the original tumor, partially accounting for the vivid reappearance of the immune checkpoint blockade through anti-PD-1- and/or anti-PD-L1 expansion, and the initiation of tumor-reactive TILs [49]. Additionally, Li et al. also employed the ALI method to achieve cancerization in a diverse gastrointestinal tissues organoid [327].

#### 4.2.5. Patient-Derived Tumor-like Cell Clusters (PTC)

In the classic co-culture PDTO system, stroma elements usually serve as latter supplements after the abandonment of original stromal cells during a prolonged passage [30], which expose the fatal deficiencies in drug assessments, like labor-intensive features and unacceptable establishment periods, and, in particular, the restrictions on ascertaining specimen origin. Currently, the research team led by Prof. Xi Jianzhong are developing a promising model, termed as PTC, through the refinement of a culture medium formula and incubation conditions. Like the primary PDTO, PTC incorporates syngeneic, epithelial, fibroblast, and immune cells derived from same specimen, which are integrated by a self-assembly mechanism. Equipped with perfectly matched stromal elements that are indispensable in PTC formation, PTC could manifest reliable patient-like responses to targeted agents or chemotherapies, with an average accuracy of 93.2% [328]. It should be emphasized that the research team have firmly described PTC as a more practical model, which is completely different from organoids, as the brief construction process ensures its commercial use for clinical decision making.

## 5. Is Co-Cultured PDTO Competent in Stroma-Related Therapy Development?

Current investigations into co-cultured PDTO persist primarily in the realm of constructing composite models, discerning alterations in phenotypes induced by stromal elements, and delving into the molecular crosstalk mechanism occurring behind the scenes. Despite their implementation for spotting stroma-related targets and the experimental-grade “drug screening”, none of these platforms have found application in commercial drug development, primarily due to their nascent developmental stage. Consequently, the following question arises: is co-cultured PDTO competent in stroma-related therapy development? To address this inquiry, this section conducts a comprehensive SWOT analysis, offering an exhaustive evaluation of the inherent potential and challenges associated with co-cultured PDTO as a stroma mimicry model for drug development, which is particularly pertinent within the context of precision medicine and personalized therapy paradigms.

### 5.1. Strengths

As an ex vivo model, the prominent asset of PDTO can be described as the devoted and persistent reflection of the genomic landscape of the primary tumors [329]. Rigorous investigations conducted within the contexts of PDAC and HNSCC have identified a consistent congruence between organoid and origin tissue among major cancer driver genes, CNV patterns, somatic single nucleotide variants and structural variants, variant allele frequency (VAF) distributions, and epigenetic profile, which suggest its promising capability in cancer biology research and drug screening, aligned with the tenets of personalized medicine [231,330]. Within the sphere of stroma emulation via co-culture, the meticulous preservation of gene expression profiles and genomic alterations contributes substantively to the nuanced and individualistic tumor–stroma crosstalk exhibited by each constituent in the biobank. This preservation not only serves as the cornerstone for personalized and inimitable bio-responses, but also captures the rich heterogeneity within isoplastic stroma elements when co-cultured with PDTO. Within a considerable cohort, each organoid can be metaphorically envisaged as an emblematic representation of the source patient [190].

Another advantage of PDTOs, setting them apart from 2D cell lines or 3D spheroids formed through forced aggregation, lies in their inherent self-organization feature and pathological progression. This unique characteristic offers biochemical and mechanical cues for tumor–stroma crosstalk, encompassing cell polarity, cytokine secretion, niche stiffness, and nutrient gradients, which guide stroma reconstruction in an in vivo pattern [224]. In alignment with the above review, Chen et al. have identified that attachment to the OSCC organoid instigated the CAF phenotype transition of para-cancerous fibroblasts, via Notch signaling. This functional activation was exemplified by the acquisition of a multiple-branch morphology in CAF, a phenomenon scarcely reported in preceding co-culture studies involving 2D cancer cells or 3D in vivo models [331].

Furthermore, a co-culture system integrating cancer organoids with specific cell types proves to be a versatile tool for diverse research objectives, exemplifying the adaptability of co-cultured PDTO in stroma research. Considering the topic of stroma-targeted therapy, the utilization of co-cultured PDTO and its application in different situations can be summarized in three points: (1) The most common use is to curtail artificial regulation during organoid formation, through direct or indirect interplay among benign and malignant cellular components within a tumor, which can be termed as crosstalk. This intricate crosstalk, a crucial determinant of organoid fate, inspires the identification of therapeutic targets associated with the stroma [190]; (2) Co-cultured PDTO facilitates the creation of a high-fidelity tumor model, especially from a stromal perspective, providing a robust experimental platform for the exploration of novel stroma-targeted therapies [332]; (3) The use of cancer organoids to generate, amplify, and estimate specific tumor-targeting cytotoxic cell or tumor-suppressive stromal cells as a countermeasure, like CAR-T cells, tumor-reactive T cells, or myo-fibroblastic CAFs [333].

Additional benefits include, but are not confined to, the judicious simplification of components within the tumor microenvironment (TME) and the accentuation of specific connections within tumor–stromal crosstalk. Furthermore, there is an endeavor to strike a delicate equilibrium between fidelity and throughput in the preliminary stages of drug screening.

### 5.2. Weaknesses

Despite advances in co-culture techniques for PDTOs, several challenges remain in applying this model to stroma-related drug development. The intricate dissection and analysis of co-culture systems, particularly those facilitating direct cellular contact, present formidable challenges when juxtaposed with mono-culture methodologies [48]. Disruptive procedures for bulk cultures, such as total protein or RNA extraction, ELISA assays, or bulk RNA-seq, lead to irreversible information loss regarding the dynamic biological changes within each cell type, resulting in a diminished cellular resolution. Moreover, the spatially relative position between different cells becomes blurred, lacking the precision to capture the nuanced interactions based on their proximity within the niche created by other cell types. The intricate interplay within this cellular landscape remains inadequately represented, necessitating a more sophisticated analytical framework for a finer-grained understanding. Additionally, the incorporation of multiple feedback loops between diverse cell types further amplifies this complexity, making the evaluation of co-culture systems a highly nuanced and challenging undertaking [334].

It is pertinent to acknowledge that, despite stromal support, achieving PDTO maturity comparable to real tumors remains challenging, due to the absence of vasculature and limited nutrient diffusion [48]. The typically small size, in the range of hundreds of micrometers, primarily reflects a single ecological niche rather than capturing broad spatial heterogeneity. Additionally, tumor-to-stroma ratios often rely on prior studies, overlooking the nuanced conditions within the tumor microenvironment [230]. As a result, organoids in pathological research often represent only a single “brick” in the larger “palace”, lacking the capacity to offer integrated insights. Reconstructing 3D tumor models through “puzzle-like” assembly remains a significant challenge in current research.

Another controversy centers around matrigel. Despite its recognition as a gold standard and a mature commodity from tumor tissue, the intricate bioactive compositions within matrigel have yet to be comprehensively investigated. This lack of thorough exploration creates a metaphorical black box, obscuring our understanding of the intricacies involved in tumor–stroma crosstalk. Concurrently, the inter-batch variations in biological products introduces a significant challenge to the reproducibility of research findings [335]. Furthermore, after solidification, matrigel undergoes a transformation, assuming the role of a barrier that hampers direct cell-to-cell contact within the co-culture system [336]. This transition adds a layer of complexity to the experimental setup, influencing the dynamic interactions between tumors and stromal components.

### 5.3. Opportunity

#### The Future Trajectory of Co-Cultured PDTO Rests Upon Strategic Combinations

Through diverse co-cultivation combinations, the potential for stromal simulation can be maximized. While the prevalent form is currently binary co-culture with CAFs [190,297], other cellular components, such as stellate cells [337], MSC [227], and osteocytes [338], can also serve as stromal supplements, tailored to specific experimental purposes. Simultaneously, organoid vascularization via co-culture with HUVEC [339], and organoid immunization through co-culture with diverse lymphocytes [340], represent additional dimensions for comprehensive TME recapitulation, which are essential for evaluating angiogenesis-targeted drugs or immune therapies. Driven by the burgeoning research on the tumor microflora, microorganism co-cultures, such as Zika virus [341] and digestive tract flora [312], have emerged as the new frontier for composite organoids. Furthermore, while binary co-culture can spotlight specific nodes within an intricate crosstalk network at a high cellular resolution, trinary or even orchestrating a co-culture system can exponentially enhance the biofidelity of stroma restoration [49]. However, it is crucial to note that, with this promotion, the analysis complexity of intercellular crosstalk also intensifies, acting as a constraint on the unbridled expansion of co-culture systems.

If diverse co-cultivation contents push the system’s established property to the utmost, then the combination with other assistive technologies profoundly expands the upper limits of co-cultured PDTO capabilities for stromal mimicry and assessment. The infusion of tissue engineering techniques into co-culture systems serves as an effective countermeasure to their inherent limitations. For instance, assembloids derived from magnetic 3D bio-printing can circumvent prolonged growth and spatial rearrangement processes among different cell types, preserving the in vivo tissue structure and pathophysiological stromal features of urothelial carcinoma [318]. In another notable example, the microfluidic tumor-on-a-chip (TOC) device not only offers precise supplement regulation opportunities, but also creates a mechanically dynamic environment, mirroring the flow shear pressure from vasculature [315]. Furthermore, addressing the analysis resolution in direct co-culture systems can be partially remedied through sophisticated cell sorting approaches, employing flow cytometry, scRNA-seq, and even mass spectrometry flow cytometry, for robust cell type identification [334].

Simultaneously, advancements in AI, particularly within the domain of deep convolutional neural networks, are propelling the automation and intelligentization of organoid observation and analysis to new heights. Notably, OrgaQuant, an end-to-end trained deep convolutional neural network tailored for 3D environments, showcases complete automation in the analysis of human intestinal organoid localization and quantification across thousands of images without any user intervention [342]. In parallel, Matthews et al. have introduced organoID, a versatile machine learning (ML) platform that is adept at tracking and detecting single-organoid dynamics in both brightfield and phase-contrast views, which maintains an impressive tracking accuracy, of over 89%. Beyond mere tracking, organoID exhibits exceptional performance when evaluating motility rates in a chemotherapy dose–response experiment, with a morphological consideration of organoid circularity, solidity, and eccentricity [343]. Within the context of therapy development, Kong et al. have devised a method for assessing drug responses in colorectal and bladder cancer backgrounds. In this research, a machine learning framework has been meticulously crafted to identify robust drug biomarkers and predict overall survival through network-based analyses employing pharmacogenomic data derived from patient-derived tumor organoids (PDTO) [344]. Undoubtedly, this promising in silico approach stands poised as a robust tool for detecting cellular interactions in co-culture systems, and facilitating the widespread adoption of organoids in high-throughput, data-intensive biomedical applications.

### 5.4. Treats

As an infant model, co-cultured PDTO requires substantial refinements before it can be confidently applied in the development of stroma-targeted therapies.

The most formidable challenge lies in the acquisition and maintenance of stromal cells. Ideally, syngeneic stromal cells derived from the same patient as the PDTO are imperative for co-culture. However, obtaining these cells poses a considerable challenge [226]. Ethical considerations aside, cell isolation imposes stringent requirements on tumor volume, as certain cell types exhibit extremely low abundance. Meanwhile, the rest of tumor specimens must simultaneously meet the criteria for organoid construction and pathological examination that guide clinical decision [345]. Addressing this challenge necessitates artificial expansion methods for stromal cells capable of preserving the original tumor-reprogrammed phenotype. Additional complexities include the identification and trace of stromal cell lineage, as primary organoid construction inevitably introduces stromal cells without sorting, enrichment, and even gene modification, based on experimental design [346].

Another pivotal concern revolves around the absence of standardization. In essence, the field lacks a universally agreed-upon cultivation criterion and a well-defined success parameter to guide the incubation of organoids, not to mention the co-culturing procedures [347]. Consequently, the diverse technical methodologies employed across different laboratories, covering aspects like specimen selection, sample quality control, tissue pretreatment and dissociation methods, routine maintenance (including passage and cryopreservation), variations in medium formulations, and the utilization of different matrices in the organoid system, contribute to fluctuations in the accurate assessment of biological heterogeneity in tumors [348]. Moreover, the standardization of fundamental reagents demands contemplation. For instance, the matrix requirements for stromal cells may deviate from cancer cells; while cancer cells may rely on intercellular interactions to evade anoikis, stromal cells typically depend on survival through integrin-mediated adhesion to the surrounding matrix [349,350]. Additionally, in comparison to standard PDTO cultivation conditions, it is imperative to optimize the growth factor combination in the medium, due to the incorporation of stromal elements as a novel variable. The fundamental debugging involves the addition of nutrients that assure not only survival, but also functional activity and the deletion of previous cytokines that can be obtained from intra-system circulation [42].

The progression of high-throughput co-cultured PDTO for efficient drug screening and personalized therapeutic testing hinges significantly upon the evolution of automation equipment and precision devices. However, the seamless integration of unmanned machinery with intricate biological systems poses formidable challenges [351]. As an example of this, the emulation of the intricacies within the TME in co-culture models necessitates the inclusion of diverse stromal cell types and sophisticated microfluidic devices. The high-throughput potential of these 3D co-culture models confronts substantial limitations due to heightened detection complexities. Consequently, there exists a critical imperative to meticulously balance the intricate nature of these models with their envisioned high-throughput capabilities. While fully automated 3D bio-printing devices have become accessible for high-content drug screening, the mechanical operations engender a notable compromise in cell viability. Furthermore, the expensive cost for equipment has led to hesitation among lots of researchers [352]. Despite the availability of user-friendly microfluidic devices facilitating the exploration of cancer cell-immune cell interactions, most of these devices are inherently constrained in scale and prove unsuitable for comprehensive drug screening applications [353]. Consequently, there emerges a compelling necessity to chart the course of future research endeavors, focusing on the development of high-throughput platforms tailored to both microfluidic devices and co-culture models.

## 6. Conclusions and Discussions

The difficulties embedded in stroma mimicry modeling can be succinctly condensed as dynamics, described in detail as inner dynamics within stroma, and mutual dynamics between stroma and neoplastic cells [159]. For the former, the stromal composition that manifests is not static complexity, but significant variability driven by either interactions within stroma or exogenous interference, pathologically depicted as stroma remodeling, an adaptation to TME alternation [354]. The latter involves intricately reciprocal tumor–stroma crosstalk, facilitating multi-dimensional heterogeneities in stroma [16]. The versatile dynamic signatures, as the essences of personalized stroma-relative treatment, threaten the delicate balance between the fidelity and feasibility of the stroma-imitation approach.

Generally, recent years have witnessed a sweeping wave of advancements across in vivo, in vitro, ex vivo, and in silico models, rendering them suitable at certain occasions during the development of therapeutics related to stroma. Despite the popular combination of 2D culture and xenograft models in the preclinical phase of stroma-targeted regimen development, the rise in ex vivo model commands sufficient attention by virtue of their capability to uphold “the simplest abstractions of reality” [213]. As the robust candidate among ex vivo models, PDTO not only preserves the personalized gene signature of the originating patient, which is essential for precise and personalized medicine, but surpasses the limitations of 2D cell culture approaches, allowing 3D cultures of multiple cell populations, unraveling cell–matrix interactions, evaluating treatment responses, and delving into the vast landscape of tumor heterogeneity [213]. When co-cultured with stromal cells, PDTO can dynamically mirror the biomimetic TME, akin to in vivo models. Synchronously, it retains the advantages of real-time interference and observation characteristic of in vitro models. Moreover, the maturity of scRNA-seq, based on PDTO, provides realistic data support for in silico modeling. Hence, as an ex vivo model, co-cultured PDTO bridges the chasm between in vivo, in vitro, and in silico models for artificially recreating tumor stroma.

The finding that deserves crucial emphasis is that, despite the superiority of co-cultured PDTO in this current review, models from four distinct dimensions have already showcased their distinctive advantages and indispensable roles, as well as their inherent deficiencies, in stroma mimicry research. This leads to a realization that there exists no one-size-fits-all model. Specific to co-cultured PDTO, inevitable limitations include gene drift during prolonged passage culture, the absence of capillary-derived microfluidic features, and the distortion in ratio and arrangement between oncocytes and stromal cells. Some of these limitations can be partially remedied by advanced tissue engineering technologies, such as digital TOC devices or assembloids generated by magnetic 3D bio-printing. In summary, a judiciously designed model system, centered around co-cultured PDTO, holds the potential to streamline this process, eventually alleviating the stringent demands on the development timeline and market entry for stroma-targeted therapy. This strategic refinement proves particularly advantageous for patients with stroma-rich or stroma-sensitive malignant tumors.

## Figures and Tables

**Table 1:** Comparison of different models’ in stroma recapitulation.

Model Type	Advantage	Disadvantage
In vivo models	Availability for intact stroma evolution process	Challenge in CAF lineage tracing
	Fidelity in pathological environment mimicry	Difficulty in precisely recognizing artificial interference and regulation related to stroma
	Containing all stromal elements (even immune system in humanized mice)	Labor-intensive model with expensive expenditure on financial and temporal aspects
	Feasibility for pharmacokinetic evaluation	Complex stroma compartment makes it difficult for real-time observation
	Detection for systemic toxicity and side effects	Unsatisfactory abundance of certain stromal elements without in vitro enrichment
	Syngeneic model allows co-injection with stromal composition	Obvious differences in stroma between rodents and mammals, as well as inevitable murine stroma evolution in PDX
	PDX maintains the personalized signatures in stroma and vasculature	Fluctuated success rate of certain modeling processes, like PDX
		Immune deficiency in xenograft model
		Numerous unknown antigen in GEMM hinder the CAR-T cell therapy assessment
In vitro models	Inherent benefits of in vitro models, including affordable expenditure, high throughput, short period and so on	Excessive simplification leads to information loss in stroma
	Rational simplification highlights interested stromal response	Minimal ECM secretion and negligible ECM accumulation
	Contrived cell ratio between tumor and stroma compared with in vivo model	Distinguishing stroma response compared with in vivo environment
	Facilitates the introduction of immune cells and immune assessment	Only support basic co-culture combinations for co-culture
	Convenience for precise interference and real-time observation	Long-term cultivation erases the personalized responses of 2D cell lines when exposed to stromal interference, making the evaluation of stromal-targeted therapies less accurate
	The high homogeneity of 2D cell lines ensures a uniform background for stroma simulation based on co-culture	2D environment results in homogenization of stromal cell
	Observable gradients of oxygen and nutrients in 3D models	Compulsory artificial structures cannot replicate the matrix compartment
	3D-printed scaffold model allows cells to be positioned in a precise and reproducible manner, as well as printing of gradients/complex shapes within the same models	Most 3D models require special equipment, for instance, digital pump for TOC, rotating flask for spheroid and 3D bioprinter for certain scaffold model
		The limited space in TOC device constrains the complexity of stroma microenvironment.
		The most common chip material, polydimethylsiloxane, possesses adsorption properties, causing interference in the development of small molecule stromal-targeted drugs
Ex vivo models	Maintains the gene signature as a suitable platform for drug development under precise medicinal context	Limited size constrains the panoramic presentation of stroma
	Affordable expenditure and short modeling period compared with PDX	Non-standardized model hides the universality of stroma environment
	Broad demand for tumor specimen, including tissue and cell from surgery, biopsy, blood, and even urine	Samples are derived from surgeries or biopsies, exhibiting a significant level of randomness and relatively low throughput
	Cell contact becomes more intimate, enhancing the efficiency of interference	Exploration and optimization are still needed for the conditional culture medium that allows a comprehensive display of stromal responses
	Observable gradients of oxygen and nutrients	TSC possesses short cultivation cycle and cannot be subcultured
	Having or supporting complex stromal cell composition	Stroma-related gene drift during prolonged passage culture of PDTO
	Individual heterogeneity in ECM accumulation and stromal response can be preserved in PDTO bio-profile	Standardized matrigel used in PDTO culture ignore the personality in ECM
	The self-organizing mechanism and pathological growth process of PDTO guarantee the cell polar arrangement and biological fidelity of tumor–stroma crosstalk	
	TSC allows the assessment of drug penetration ability	
In silico models	Data and algorithm accumulation exponentially magnify the capability of stroma recapitulation	Stringent demand for bioinformatics techniques
	Virtual form is convenient for interplay and sharing	Satisfied repeatability for stroma imitation only stays in the dry lab stage
	Impressive efficiency allows large volume of stroma-targeted drug selection or design simultaneously	Subjectivity in dataset selection may cause bias in stroma reappearance in silico
	Expansion for research, including stroma evolution and remodeling, tumor–stroma crosstalk, biomarker detection in stroma, drug-related stroma response and stroma-related prognostic prediction.	Sequencing data generated at a certain moment, causing difficulty for continue observation targeting dynamic stroma change
	Computational model can achieve ultramicro or ultramacro scale simulation to stroma response that is difficult in realistic model	
